# CRISPR-act-mediated synchronized activation of *CsAlaDC* and *CsTSI* enhances L-theanine biosynthesis in tea plants

**DOI:** 10.1093/hr/uhag028

**Published:** 2026-01-29

**Authors:** Xuening Zhang, Qianyuan Fu, Xiaobin Dong, Yan Shen, Yuwan Hao, Ying Yang, Jialu Fang, Meng Ye, Changqing Ding, Xinyuan Hao, Lu Wang, Nana Li, Jianming Zeng, Xinchao Wang, Jianyan Huang

**Affiliations:** Key Laboratory of Biology, Genetics and Breeding of Special Economic Animals and Plants, Ministry of Agriculture and Rural Affairs, State Key Laboratory of Tea Plant Germplasm Innovation and Resource Utilization, Tea Research Institute, Chinese Academy of Agricultural Sciences, Hangzhou 310008, China; Key Laboratory of Biology, Genetics and Breeding of Special Economic Animals and Plants, Ministry of Agriculture and Rural Affairs, State Key Laboratory of Tea Plant Germplasm Innovation and Resource Utilization, Tea Research Institute, Chinese Academy of Agricultural Sciences, Hangzhou 310008, China; Key Laboratory of Biology, Genetics and Breeding of Special Economic Animals and Plants, Ministry of Agriculture and Rural Affairs, State Key Laboratory of Tea Plant Germplasm Innovation and Resource Utilization, Tea Research Institute, Chinese Academy of Agricultural Sciences, Hangzhou 310008, China; Key Laboratory of Biology, Genetics and Breeding of Special Economic Animals and Plants, Ministry of Agriculture and Rural Affairs, State Key Laboratory of Tea Plant Germplasm Innovation and Resource Utilization, Tea Research Institute, Chinese Academy of Agricultural Sciences, Hangzhou 310008, China; Key Laboratory of Biology, Genetics and Breeding of Special Economic Animals and Plants, Ministry of Agriculture and Rural Affairs, State Key Laboratory of Tea Plant Germplasm Innovation and Resource Utilization, Tea Research Institute, Chinese Academy of Agricultural Sciences, Hangzhou 310008, China; Key Laboratory of Biology, Genetics and Breeding of Special Economic Animals and Plants, Ministry of Agriculture and Rural Affairs, State Key Laboratory of Tea Plant Germplasm Innovation and Resource Utilization, Tea Research Institute, Chinese Academy of Agricultural Sciences, Hangzhou 310008, China; Key Laboratory of Biology, Genetics and Breeding of Special Economic Animals and Plants, Ministry of Agriculture and Rural Affairs, State Key Laboratory of Tea Plant Germplasm Innovation and Resource Utilization, Tea Research Institute, Chinese Academy of Agricultural Sciences, Hangzhou 310008, China; Key Laboratory of Biology, Genetics and Breeding of Special Economic Animals and Plants, Ministry of Agriculture and Rural Affairs, State Key Laboratory of Tea Plant Germplasm Innovation and Resource Utilization, Tea Research Institute, Chinese Academy of Agricultural Sciences, Hangzhou 310008, China; Key Laboratory of Biology, Genetics and Breeding of Special Economic Animals and Plants, Ministry of Agriculture and Rural Affairs, State Key Laboratory of Tea Plant Germplasm Innovation and Resource Utilization, Tea Research Institute, Chinese Academy of Agricultural Sciences, Hangzhou 310008, China; Key Laboratory of Biology, Genetics and Breeding of Special Economic Animals and Plants, Ministry of Agriculture and Rural Affairs, State Key Laboratory of Tea Plant Germplasm Innovation and Resource Utilization, Tea Research Institute, Chinese Academy of Agricultural Sciences, Hangzhou 310008, China; Key Laboratory of Biology, Genetics and Breeding of Special Economic Animals and Plants, Ministry of Agriculture and Rural Affairs, State Key Laboratory of Tea Plant Germplasm Innovation and Resource Utilization, Tea Research Institute, Chinese Academy of Agricultural Sciences, Hangzhou 310008, China; Key Laboratory of Biology, Genetics and Breeding of Special Economic Animals and Plants, Ministry of Agriculture and Rural Affairs, State Key Laboratory of Tea Plant Germplasm Innovation and Resource Utilization, Tea Research Institute, Chinese Academy of Agricultural Sciences, Hangzhou 310008, China; Key Laboratory of Biology, Genetics and Breeding of Special Economic Animals and Plants, Ministry of Agriculture and Rural Affairs, State Key Laboratory of Tea Plant Germplasm Innovation and Resource Utilization, Tea Research Institute, Chinese Academy of Agricultural Sciences, Hangzhou 310008, China

Dear Editor,

L-theanine is a unique nonproteinaceous amino acid predominantly synthesized in the roots of tea plants (*Camellia sinensis*) [1]. While the completion of the tea plant genome has accelerated research into key metabolic pathways such as L-theanine biosynthesis, the functional genomics toolkit for tea plants remains underdeveloped. Previous studies have pioneered the use of CRISPR/Cas9 for genome editing in tea plants, successfully targeting genes in the L-theanine and caffeine pathways [2, 3]. However, these foundational efforts have primarily relied on generic, heterologous promoters (e.g., from *Arabidopsis*) without a systematic evaluation of their performance in tea plants. Furthermore, powerful gain-of-function tools, such as the CRISPR-Act system [4], have yet to be implemented. Here, we address these critical gaps by identifying and validating superior endogenous promoters for robust CRISPR applications in tea plants, and establishing the first CRISPR-Act system for synchronized, multigene activation to significantly boost theanine synthesis.

To identify efficient promoters for sgRNA and Cas9 expression, we first isolated four CsU3 and eight CsU6 promoter candidates from the *Camellia sinensis* cv. ‘Longjing 43’ genome based on sequence homology to AtU3 and AtU6 (Fig. S1). The transcriptional activities of these promoters were evaluated in *Nicotiana benthamiana* via a GUS-based transient expression assay. The results showed that two U3 promoters, CsU3a and CsU3b, exhibited significantly higher transcriptional activity than the AtU3 control (Fig. 1A and B). Among the eight CsU6 promoters, CsU6i and CsU6j showed the highest activity, although this was still lower than that of AtU6 (Fig. 1A and C). Notably, AtU6 activity was approximately 39% lower than that of AtU3 in this system. Consequently, we selected AtU3 as the high-stringency benchmark control for subsequent comparisons. For Cas9 expression, we identified four *CsUBI* candidates (*CsUBI1*, *2*, *10a*, and *10b*) based on homology to the constitutive *Arabidopsis* genes (*AtUBI1/2/10*, Fig. S2). An initial assessment in the same tobacco system revealed negligible activity for *CsUBI1* (Fig. 1D). To rule out heterologous expression artifacts, we further evaluated all four candidates in homologous tea plant protoplasts. Dual-luciferase assays confirmed that all endogenous *CsUBI* promoters were significantly weaker than the CaMV 35S promoter (Fig. 1E). Therefore, the 35S promoter was selected to drive Cas9 expression in the gene editing vectors for all subsequent experiments.

**Figure 1:**
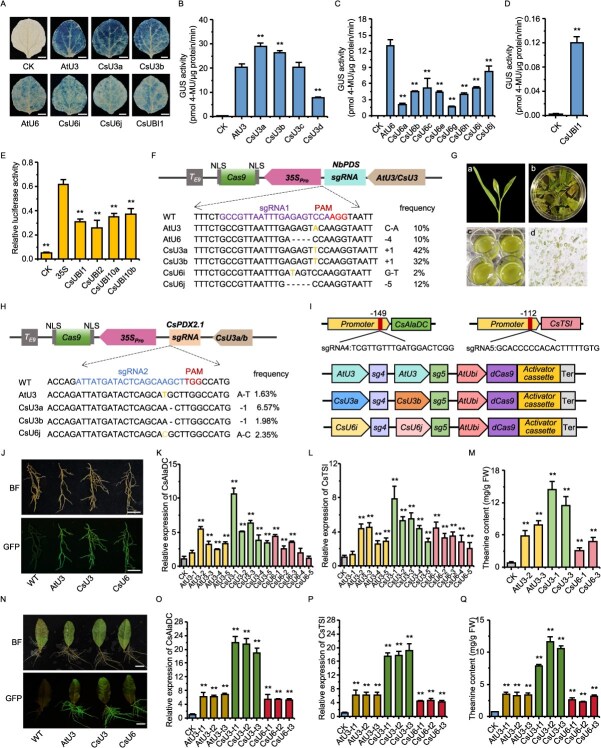
Establishing a CRISPR-Act system for theanine enhancement in tea plants. (A) Histochemical GUS staining of constructs driven by CsU3, CsU6, and CsUBI promoters in tobacco leaves. AtU3 and AtU6 were used as controls. Scale bar = 1 cm. (B-D) Relative transcriptional activity of CsU3 (B), CsU6 (C), and CsUBI (D) promoters assessed by GUS reporter assay in tobacco. In these panels, CK indicates empty vector-transformed tobacco. (E) Transcriptional activity of CsUBI promoters in tea plant protoplasts using the Dual-Luciferase reporter system. The 35S promoter was used as control. CK represents the background relative LUC activity in untransformed protoplasts. (F) Editing efficiencies of different promoter-driven CRISPR/Cas9 constructs at the *NbPDS* target site in tobacco. The frequency was calculated as the ratio of edited to transformed leaves (n = 50). (G) Schematic representation of tea plant protoplast isolation and transformation. Panels a-d show: (a) experimental materials, (b) enzymatic digestion, (c) protoplast purification, and (d) protoplasts after PEG-mediated transformation. (H) Editing efficiencies of different promoter-driven CRISPR/Cas9 constructs at the *CsPDX2.1* target site in tea protoplasts. (I) Schematic diagram of the CRISPR-Act3.0 vector used for activating endogenous genes in tea plant, with sgRNA driven by various U3 or U6 promoters. (J) Representative image of GFP-positive tea roots after *A. rhizogenes*-mediated transient transformation. Scale bar = 0.5 cm. (K, L) Relative expression levels of *CsAlaDC* (K) and *CsTSI* (L) in transiently transformed tea roots driven by different promoters. Gene expression was normalized to an internal control gene *CsPTB* and compared to the empty vector control (CK) (M) L-theanine content in transiently transformed tea plant roots (CK: empty vector control). (N) Induction of stable hairy roots from tea leaf explants after two months of culture. Scale bar = 2 cm. (O, P) Relative expression levels of *CsAlaDC* (O) and *CsTSI* (P) in stable hairy root lines driven by different promoters. (Q) L-theanine content in stable hairy root lines (CK: empty vector control). In all panels, error bars represent the standard deviation (n = 3), and asterisks denote significant differences as determined by Student’s *t*-test (^**^P < 0.01).

To evaluate the functional efficacy of the selected sgRNA promoters, we constructed a series of CRISPR/Cas9 vectors. We first validated these constructs by targeting the *NbPDS* gene in tobacco. All six promoter-driven constructs successfully induced mutations, with CsU3a and CsU3b exhibiting notably high efficiencies of 42% and 32%, respectively (Fig. 1F). Encouraged by this, we further tested the constructs in tea plant protoplasts targeting the *CsPDX2.1* locus (Fig. 1G). High-throughput sequencing revealed that CsU3a was the most effective promoter (6.57% efficiency), outperforming CsU6j (2.35%), CsU3b (1.98%) and the AtU3 control (1.63%) (Fig. 1H). However, no edits were detected with CsU6i-driven constructs. These results demonstrate that the endogenous CsU3 promoters are capable of driving efficient gene editing in tea plants, correlating with their higher transcriptional activity observed in the reporter assays.

As an alternative to traditional ectopic overexpression, we employed the CRISPR-Act3.0 system to upregulate endogenous genes in the L-theanine pathway. We targeted two key genes, *CsAlaDC* and *CsTSI* [5], with sgRNA expression driven by AtU3 and the most active promoters identified from each of the CsU3 and CsU6 families (Fig. 1I). In *Agrobacterium rhizogenes*-mediated transient root assays (Fig. 1J), expression of *CsAlaDC* and *CsTSI* was upregulated by up to 10.5-fold using the CsU3-driven construct (Fig. 1K and L), leading to a ~5.8-fold increase in L-theanine content (Fig. 1M). However, variability was observed in several samples (e.g., AtU3-1, CsU6-4, CsU6-5), likely due to the inherent instability of transient expression.

To overcome the expression variability inherent in conventional transient transformations, we inoculated tea leaves with *A. rhizogenes* harboring the CRISPR-Act plasmid and cultured the infected leaves in sterile vermiculite for hairy root induction. After approximately two months, hairy roots developed successfully from the petiole bases of several explants (Fig. 1N). Roots displaying uniform fluorescence were excised and subjected to qRT-PCR analysis. The results showed that the expression levels of both *CsAlaDC* and *CsTSI* were significantly upregulated: by 6.4-fold with AtU3, 20.8-fold with CsU3, and 5.3-fold with CsU6 (Fig. 1O and P). Correspondingly, L-theanine contents were elevated by 3.8-fold (AtU3), 8.5-fold (CsU3), and 2.6-fold (CsU6), respectively (Fig. 1Q). In parallel, we observed a mild elevation of alanine levels in specific lines (e.g., CsU3-1) and a notable increase in ethylamine levels across most CRISPR-Act lines (Fig. S3). These findings demonstrate that our CRISPR-Act system, particularly when driven by the endogenous CsU3 promoter, can robustly and stably co-activate multiple genes to enhance L-theanine biosynthesis in tea plants.

In summary, our study provides the first demonstration of synchronized activation of endogenous genes in tea plants using the CRISPR-act system. We identified and validated a suite of native CsU3 promoters that efficiently drive both CRISPR-based gene editing and activation. This work provides a powerful and versatile toolkit for functional genomics and metabolic engineering in tea plants.

## Data Availability

The data supporting the findings of this study have been deposited in ScienceDB and are accessible at https://doi.org/10.57760/sciencedb.34912.
